# 6-year follow-up of 84 patients with cartilage defects in the knee: Knee scores improved but recovery was incomplete

**DOI:** 10.3109/17453674.2010.519166

**Published:** 2010-10-08

**Authors:** Sverre Løken, Stig Heir, Ingar Holme, Lars Engebretsen, Asbjørn Årøen

**Affiliations:** ^1^Orthopaedic Department, Oslo University Hospital, Oslo, Norway; ^2^Martina Hansens Hospital, Oslo, Norway; ^3^Oslo Sports Trauma Research Center, Oslo, Norway

## Abstract

**Background and purpose:**

The natural history of focal cartilage injury is largely unknown. In this study we investigated 6-year outcomes in patients with arthroscopically verified, focal, full-thickness cartilage injuries of the knee.

**Methods:**

In a previous report (baseline study) of 993 knee arthroscopies, 98 patients were less than 50 years old at baseline and showed grade 3–4 focal cartilage injury, as assessed with the International Cartilage Repair Society (ICRS) scale. In the present study, 84 of the 98 patients completed follow-ups at median 6.1 (5.3–7.8) years after baseline assessments. At baseline, the patients had undergone different types of cartilage repair (n = 34) or had no treatment or only debridement (n = 64) for their cartilage injury. The follow-up included evaluations with the ICRS knee evaluation form, the Lysholm score, and other knee evaluation tests. 68 patients underwent radiographic assessments with weight bearing.

**Results:**

Improvements compared to baseline were noted in the average ICRS functional score, visual analog scale (VAS) pain score, and the patients' rating of the function in the affected knee compared to the contra-lateral knee. However, the average ICRS activity level had decreased from baseline. The average Lysholm score was 76 (SD 21). 19 patients had Kellgren-Lawrence grades 2–3 in the affected knee and 6 patients had grades 2–3 in the contralateral knee. There was a statistically significant difference between affected and contralateral knees.

**Interpretation:**

Patients with arthroscopically diagnosed ICRS grade 3–4 cartilage injuries in the knee may show improvement in knee function over the following 5–8 years, with or without cartilage repair. However, knee function remains substantially affected. Further studies are needed to determine whether cartilage surgery can yield better functional outcomes than non-surgical or less invasive surgical treatments.

Focal cartilage and osteochondral injuries in the knee are common (Hjelle et al. 2002, Årøen et al. 2004, Widuchowski et al. 2007) and may cause impairments in quality of life similar to those associated with severe osteoarthritis (OA) (Heir et al. 2009). The natural history of focal cartilage injuries is largely unknown. For example, it is not clear whether cartilage injury always leads to some degree of OA or whether there is a critical size or depth of cartilage injury that progresses to OA. Cartilage injuries are commonly associated with anterior cruciate ligament (ACL) injuries. Data from the Norwegian National Cruciate Ligament Registry show that preoperative Knee Injury and Osteoarthritis Outcome Scores (KOOS) in patients who have an ACL injury with an associated cartilage injury are similar to those in patients who have an ACL injury with no associated cartilage injury (Hjermundrud et al. 2010), indicating that cartilage injuries do not always affect knee function. Although many cartilage injuries are asymptomatic, several randomized controlled trials on the repair of chronic focal cartilage injuries have shown that cartilage injuries may lead to severe disability (Bentley et al. 2003, Horas et al. 2003, Knutsen et al. 2004, Gudas et al. 2005, Knutsen et al. 2007, Saris et al. 2008, 2009). Taken together, these studies have not demonstrated that one method is superior to any other, but none have included a control group of patients that did not undergo cartilage repair. The best outcomes have been reported after autologous chondrocyte implantation (ACI) in young, active patients with a high preoperative score, a single defect, and less than 2 years of symptoms (Krishnan et al. 2006). Due to the lack of knowledge concerning the natural history of cartilage injuries, we performed a 6-year follow-up study on a group of patients with a cartilage injury found at arthroscopy. Our study hypothesis was that patients with an arthroscopically diagnosed cartilage injury would not show any change in knee function at the 6-year follow-up compared to baseline.

## Patients and methods

The patients in this follow-up study were part of a group of patients from a previous report (baseline study) of 993 knee arthroscopies (Årøen et al. 2004) (Figure). This follow-up study included the following inclusion criteria: a cartilage lesion classified as a focal ICRS (International Cartilage Repair Society 1998) grade 3–4 injury, and less than 50 years old at the time of arthroscopy in the baseline study. Both traumatic and osteochondritis dissecans (OCD) lesions were included. Patients classified as having general osteoarthritis either by radiography or at arthroscopy were excluded. 98 patients fulfilled the inclusion criteria. Of these, 2 patients had died; thus, the remaining 96 patients were asked to participate in the follow-up study. 5 patients were lost to follow-up. 7 patients responded, but did not attend the follow-up examination and did not return the questionnaires after repeated contact by letter and telephone. 84 patients completed the questionnaires at median 6.1 years (range 5.3–7.8) after the baseline arthroscopy. Of these, 77 patients attended a physical examination and 7 returned the questionnaires by mail. 68 patients completed the radiographic examination.

**Figure:**
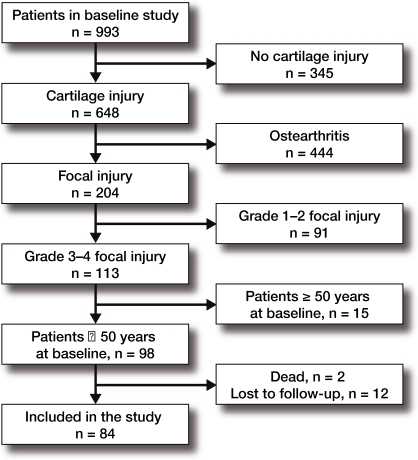
Flow chart showing the selection of patients from the baseline study to the follow up study.

The patients were divided into 4 subgroups according to the procedures that were performed at baseline (Table 1). The surgical procedures stated were performed as a part of the arthroscopic procedure. Patients undergoing a cartilage biopsy for cell culture before autologous chondrocyte implantation (ACI) were described as being treated with ACI at baseline, as the arthroscopy in this case was part of a two-stage procedure. Group 1 (n = 25) had no cartilage repair and no ligament/meniscal surgery performed at baseline. Of these patients, 13 underwent a diagnostic knee arthroscopy only, 7 underwent debridement, 3 underwent a lateral retinacular release, 1 underwent fixation of a fracture, and 1 underwent removal of an implant. Group 2 (n = 39) did not undergo cartilage repair, but did undergo ligament and/or meniscal surgery at baseline. Group 3 (n = 21) underwent cartilage repair at baseline but did not undergo additional ligament/meniscal surgery. Group 4 (n = 13) underwent both cartilage repair and additional ligament/meniscal surgery at baseline. Cartilage repair was defined as ACI, microfracture, osteochondral cylinder transfer (OCT), or fixation of an osteochondral fragment. These procedures, together with a postoperative rehabilitation program, were aimed at inducing repair of the cartilage defect. The aim of debridement of a cartilage lesion was to reduce mechanical symptoms and/or inflammation, and it was therefore not considered a cartilage repair.

**Table 1.** Baseline characteristics of patients with knee cartilage defects. Patients were grouped based on the types of procedures performed

	Subgroup 1	Subgroup 2	Subgroup 3	Subgroup 4	All patients
Cartilage repair: **^a^**	No	No	Yes	Yes	
Ligament/meniscus surgery:	No	Yes	No	Yes	
	(n = 25)	(n = 39)	(n = 21)	(n = 13)	(n = 98)**^b^**
Baseline characteristics: mean (SD)					
Age at start of symptoms	25 (10)	31 (11)	25 (11)	32 (8.9)	28 (11)
Age at baseline	28 (9.4)	35 (9.4)	30 (10)	35 (8.6)	32 (9.7)
BMI at baseline	24 (4.4)	25 (3.8)	24 (3.3)	26 (4.5)	25 (3.9)
Females/males	12/13	14/25	8/13	4/9	38/60
Area of cartilage defect**^c^** (no. of patients)					
< 1 cm^2^	3	7	0	1	11
1–2 cm^2^	8	18	6	0	32
2–4 cm^2^	6	6	6	6	24
> 5 cm^2^	8	8	9	6	31
Total	25	39	21	13	98
Location of cartilage defects**^d^** (no. of defects)					
Lateral femoral condyle	5	5	2	5	17
Medial femoral condyle	9	21	17	8	55
Lateral tibial condyle	4	5	2	0	11
Medial tibial condyle	4	2	0	2	8
Patella	10	14	3	1	28
Total	32	47	24	16	119 **^e^**

**^a^** Cartilage surgery was defined as: an autologous chondrocyte implantation (with a preceding biopsy), an osteochondral cylinder transfer, a microfracture repair, or fixation of osteochondral fragment.

**^b^** All patients fulfilling the inclusion criteria were included in the table (n = 98). Of these, 2 had died, 12 were lost to follow-up, and 84 were included in the final follow-up.

**^c^** In patients with more than one defect, the areas given represent the sum of the areas.

**^d^** All injuries were rated as grade 3 or 4 on the International Cartilage Repair Society scale.

**^e^** Some patients had more than one defect; thus, the total number of defects exceeded the number of patients.

In the baseline study (Årøen et al. 2004), assessments were based on the first version of the ICRS form (International Cartilage Repair Society 1998). Thus, the same version was used in this follow-up study to facilitate comparisons.

The baseline characteristics of the patients are given in Table 1. The ICRS form included a history of previous surgeries to the knee. This information was supplemented with records of surgery from our hospital and other hospitals. Surgical procedures that were performed before baseline, at baseline, and after baseline are shown in Table 2.

**Table 2.** The numbers of patients with knee cartilage defects who underwent the procedures shown. Patients were grouped based on the types of procedures performed at baseline. In some cases, more than one procedure was performed in a single knee; thus, the number of procedures exceeds the number of patients

	Subgroup 1			Subgroup 2			Subgroup 3			Subgroup 4			All patients		
Cartilage repair: **^a^**	No			No			Yes			Yes					
Ligament/meniscus surgery:	No			Yes			No			Yes					
	(n = 25)			(n = 39)			(n = 21)			(n = 13)			(n = 98) **^b^**		
Time of procedures: **^c^**	B	BL	A	B	BL	A	B	BL	A	B	BL	A	B	BL	A
Cartilage repair:															
Microfracture	1						1	11	1	1	10	2	3	21	3
Osteochondral cylinder transfer			1					2	1					2	2
Biopsy preceding ACI **^d^**			1			1		4	1		3	1		7	4
Fixation of osteochondral fragment								4						4	
Other procedures:															
Diagnostic arthroscopy	6	13	3	7	6		9			2			24	19	3
Debridement of cartilage injury		7	1		3	1			3			1		10	6
Ligament reconstruction	1			2	10	1	1			2	2	2	6	12	3
Meniscal resection	1			7	24	2			2	1	5		9	29	4
Other	2	5	3			2			1			1	2	5	7
No information	3			2									5		

**^a^** Cartilage repair was defined as: an autologous chondrocyte implantation (with a preceding biopsy), an osteochondral cylinder transfer, microfracture, or fixation of osteochondral fragment.

**^b^** All patients fulfilling the inclusion criteria were included in the table (n = 98). Of these, 2 had died, 12 were lost to follow-up, and 84 were included in the final follow-up.

**^c^** The time the procedures were performed, expressed relative to the baseline assessment. B: before baseline; BL: at baseline; A: after baseline (during follow-up).

**^d^** ACI: autologous chondrocyte implantation.

The primary outcome variable was the change in functional status from baseline according to the ICRS form. Secondary outcome variables were change in the activity level and change in the patient's rating of the affected knee compared to the contralateral knee. The ICRS questionnaire also included a visual analog scale (VAS), where zero represented a pain-free joint and 100 represented “severe pain”. Changes in the outcome variables over time were calculated for all patients and for the subgroups.

Other questionnaires used at follow-up included the Tegner score (Tegner and Lysholm 1985), the Lysholm score (Lysholm and Gillquist 1982), the KOOS (Roos et al. 1998), the International Knee Documentation Committee's subjective knee form (IKDC) (Hefti et al. 1993, Irrgang et al. 2001), the Cincinnati knee rating system (Noyes et al. 1983), and the short-form 36 health survey (SF-36) (Ware and Sherbourne 1992). Both the Lysholm score (Kocher et al. 2004) and the KOOS score (Bekkers et al. 2009) have been validated for patients with cartilage injuries. The Tegner and Lysholm scores are not completely self-explanatory; thus, an examiner provided guidance for the patients as they filled out the form. The other forms were filled out by the patients without any guidance.

According to the ICRS form, the patients did single-leg length jumps on the right and left legs (completed by 59 patients). The average length of 3 jumps was calculated for each leg. The average jump distance achieved with the affected leg was expressed as a percentage of the average jump distance performed with the contralateral leg as follows: affected leg function = (average jump length with affected leg / average jump length with contralateral leg) × 100. The results were classified into 4 categories as follows: 1. > 90%; 2. 76–90%; 3. 50–75%; and 4. < 50%.

Weight bearing radiographs were performed according to a routine protocol: anteroposterior view with extended knees and lateral view. The radiographs were examined by one of the authors (LE) who was blinded regarding the affected side, and they were classified according to the criteria of Kellgren and Lawrence (Kellgren and Lawrence 1957).

### Statistics

We used Wilcoxon's paired rank test for assessment of changes in the following variables from baseline: the ICRS functional level, the ICRS activity level, and the ICRS rating of the knee compared to the contralateral knee. All these were categorical variables with 4 levels (1–4); patients served as their own controls and p-values were adjusted for tied observations. A paired t-test was used for analysis of VAS pain scores. For each outcome (dependent) variable, a linear regression model was fitted to determine whether the following factors were predictors: area of the cartilage lesion, location of the cartilage lesion, cartilage repair performed at baseline, and ligament or meniscal surgery performed at baseline. After univariate and multivariate analyses of these factors, additional adjustment for the following potential confounding factors was performed in a multivariate analysis: age at the start of symptoms, age at baseline, sex, and body mass index (BMI). For functional assessments, we regarded an average difference of 0.5 units (17%) on the 1–4 functional level scale as a difference of clinical interest. A priori sample size calculation showed that 47 patients would be sufficient to be able to detect a 0.5-unit average change in functional level from baseline with a standard deviation of 1.2 units, with 80% power, and with a 5% significance level.

Radiographic assessments of affected and contralateral legs were compared with a Wilcoxon paired rank test. We used SPSS version 15.0 for the statistical analysis. For all analyses, the significance level was set to 5%.

### Ethics

The study had a non-interventional design and was approved by the regional ethical committee (date of issue: October 11, 2004; registration number: 517-04197). All patients provided informed written consent to participate.

## Results

### Change from baseline (ICRS form)

At the follow-up, patient assessments indicated that on average the functional level increased, the VAS pain score decreased, the rating of knee function compared to the contralateral knee increased, and the activity level decreased compared to baseline (Table 3). All these changes were statistically significant (p ≤ 0.001). All subgroups reported improvements in functional levels and in VAS pain scores, but the change was not significant in the smallest group (cartilage repair + ligament/meniscal surgery). All subgroups reported improvements in knee function compared to the contralateral knee. All subgroups reported a reduction in activity level, but the reduction was statistically significant only in the largest group (no cartilage repair, but with ligament/meniscal surgery).

**Table 3.** Knee function assessments based on ICRS questionnaires at baseline and follow-up. Patients were grouped based on the types of procedures performed. Values indicate the numbers of patients in each subgroup who agreed with the corresponding item

	Subgroup 1		Subgroup 2		Subgroup 3		Subgroup 4		All patients	
Cartilage repair: **^a^**	No		No		Yes		Yes			
Ligament/meniscus surgery:	No		Yes		No		Yes			
	(n = 19/25)		(n = 35/39)		(n = 18/21)		(n = 12/13)		(n = 84/98) **^b^**	
	BL **^b^**	FU **^b^**	BL	FU	BL	FU	BL	FU	BL	FU
Functional level										
I can do everything I want do with my knee	1	6	0	6	0	4	0	1	1	17
I can do nearly everything I want to do with my knee	4	6	5	19	2	8	1	4	12	37
I am restricted and a lot of things that I want to do with my knee are not possible	8	5	20	10	10	6	7	4	45	25
I am very restricted and I can do almost nothing with my knee without severe pain and disability	9	2	11	0	7	0	4	3	31	5
Total	22	19	36	35	19	18	12	12	89	84
P-value (Wilcoxon test)	0.05		< 0.001		0.002		0.096		< 0.001	
Activity level										
Are you a highly competitive sportsman/sportswoman?	3	1	9	4	6	1	6	2	24	8
Are you well trained and frequently participate in sport?	10	5	14	9	5	6	1	2	30	22
Participate in sport sometimes	8	10	10	17	5	9	4	5	27	41
Never participate in sport	2	3	3	4	4	2	2	3	11	12
Total	23	19	36	34	20	18	13	12	92	83
P-value (Wilcoxon test)	0.05		0.03		0.2		0.2		0.001	
Patient's rating of knee function in comparison with the contralateral knee										
90–100%	1	8	1	18	1	7	0	4	3	37
70–90%	5	4	10	15	3	7	0	3	18	29
40–70%	8	4	19	2	10	1	9	2	46	9
0–40%	9	2	5	0	5	2	3	1	22	5
Total	23	18	35	35	19	17	12	10	89	80
P-value (Wilcoxon test)	0.009		< 0.001		0.009		0.03		< 0.001	
VAS Pain ^**c**^										
Mean values	56	34	44	24	51	32	46	31	48	29
SD	23	29	21	19	23	23	24	23	22	23
Change in VAS pain	22		21		19		15		20	
SD	38		25		13		25		25	
95% confidence intervaI	0.4–44		12–30		12–26		-2.6–33		14–26	
P-value (paired t-test)	0.05		< 0.001		< 0.001		0.09		< 0.001	

**^a^** Cartilage repair was defined as: an autologous chondrocyte implantation (with a preceding biopsy), an osteochondral cylinder transfer, microfracture, or fixation of osteochondral fragment.

**^b^** Refers to the number of patients who attended follow-up (FU) in relation to the number of patients at baseline (BL).

**^c^** VAS: visual analog scale with zero representing a pain-free joint and 100 representing “severe pain”.

### Other test scores at follow-up

All the knee-specific scores (the KOOS, Lysholm, Tegner, IKDC, Cincinnati knee rating system, SF-36, and the single-leg length jump) showed average knee function in the range of 61–86% of the maximum score for the total group, with the KOOS quality of life and sport/recreation being mostly affected and the KOOS activities of daily life being least affected (Table 4, see supplementary data). The average Tegner score was 4.3 (SD 1.9), which corresponded to a moderate level of non-pivoting sports. The physical components of the SF-36 general health score were in the range of 71–83% of the maximum score.

### Linear regression analysis

In univariate and multivariate analysis, for the primary outcome variable (change in ICRS functional level) there was no predictive association with the area or location of the cartilage lesion, cartilage repair performed at baseline, or ligament or meniscal surgery performed at baseline (Table 5, see supplementary data). Adjustment for age at the start of symptoms, age at operation, sex, and BMI did not alter these findings. The same result was found for all outcome variables including Lysholm score, Cincinnati score, IKDC, KOOS, and SF-36 (data not shown). An association was found between BMI and the Kellgren-Lawrence grade of the operated knee (multivariate analysis, p = 0.002).

### Radiographs

Radiographs were obtained from 68 patients. The difference between the operated knee and the contralateral knee was statistically significant in the total group of patients and in subgroups 1, 2, and 3 (Table 6). No significant differences were found between operated knees between all subgroups (Kruskal-Wallis non-parametric test for several independent samples) or between operated knees in pairs of subgroups (Mann-Whitney non-parametric U-test for 2 independent samples).

**Table 6.** The numbers of patients with knee cartilage defects graded 0–3 on radiographs at follow-up. Weight bearing radiographs were graded according to Kellgren and Lawrence. Patients were grouped based on the types of procedures performed

	Subgroup 1		Subgroup 2		Subgroup 3		Subgroup 4		All patients	
Cartilage repair: **^a^**	No		No		Yes		Yes			
Ligament/meniscus surgery:	No		Yes		No		Yes			
	(n = 15/25)		(n = 27/39)		(n = 17/21)		(n = 9/13)		(n = 68/98) **^b^**	
	O **^c^**	CL**^c^**	O	CL	O	CL	O	CL	O	CL
Grade 0	10	15	8	22	8	15	3	5	29	57
Grade 1	2		9	4	6		3	1	20	5
Grade 2	1		8	1	3	2	2	1	14	4
Grade 3	2		2				1	2	5	2
P-value **^d^**	0.04		< 0.001		0.03		0.8		< 0.001	

**^a^** Cartilage repair was defined as: an autologous chondrocyte implantation (with a preceding biopsy), an osteochondral cylinder transfer, microfracture, or fixation of osteochondral fragment.

**^b^** Refers to the number of patients with radiographs at follow-up in relation to the number of patients at baseline.

**^c^** O: Operated knee; CL: Contra-lateral knee.

**^d^** Wilcoxon test for difference between operated and contralateral knee

## Discussion

We found improvements in ICRS functional knee scores over time. In a linear regression analysis, we did not detect any association between the type of surgery (including cartilage repair) and the functional outcome. However, these results must be interpreted with caution because they were averages from a mixed patient cohort. The most common cartilage repair procedure performed in our patients was microfracture; thus, the results may not be generally applicable to all cartilage repair patients. Our study was not originally designed to compare different treatment procedures. Groups 3 and 4 had a higher proportion of lesions that were larger than 2 cm^2^ than groups 1 and 2. Moreover, most cartilage lesions were patellar in groups 1 and 2. Consequently, the groups should not be compared; each should be evaluated separately. The Lysholm score (average 76) and Cincinnati score (average 71) were low at follow-up; this indicated that cartilage defects had strong effects on knee function, irrespective of treatment. Another study from our group on other patient cohorts has confirmed this observation (Heir et al. 2010).

The improvements observed in knee scores may have been due to a real improvement, to the various therapeutic procedures performed, to a placebo effect of surgery, and/or to a favorable natural history. Another explanation for the observed improvement in the functional score and the reduction in pain rating on the VAS scale may have been that the patients reduced their activity levels over the years after the initial surgery. With less physical activity, there may have been less pain.

Most studies that have evaluated outcomes after cartilage surgery have reported improvements in functional scores, both in case series and in randomized controlled trials (RCTs). In a review of studies that evaluated outcomes after cartilage repair (Jakobsen et al. 2005), Lysholm scores were reported in 17 of the 61 studies included in the review, with 95% confidence intervals of 78–97 for microfracture, 86–95 for OCT, and 67–99 for ACI, with no statistically significant differences between the treatment modalities. Results from 2 RCTs that evaluated Lysholm scores (Horas et al. 2003, Knutsen et al. 2007) and from 1 RCT that evaluated KOOS scores (Saris et al. 2008, 2009) after cartilage surgery showed mean scores that were in the same range at follow-up as those found in our study. Results from 2 other RCTs could not be compared with our data. One, by Bentley et al. (2003), used the Cincinnati score but categorized the patients into groups without giving any mean values; the other, by Gudas et al. (2005), used the ICRS questionnaire without reporting their methods for the calculations. Thus, the patients in our study had an average functional level similar to that of patients who had undergone cartilage surgery in RCTs that gathered similar data. However, patients in RCTs must fulfill strict inclusion criteria; thus, those results may not be applicable to all patients with cartilage injuries (Engen et al. 2009). Nevertheless, conclusions from RCTs are often generalized to all patients with cartilage injuries. One strength of our study was that it represented the average patient with a cartilage injury who is seen in orthopedic practice.

The Lysholm score found in the present study was in the same range as that found in patients with ACL injury who were awaiting surgery (Drogset et al. 2005). The KOOS scores in the present study were slightly higher than preoperative KOOS scores in the Norwegian National Knee Ligament Registry (Granan et al. 2008), but they were still clearly abnormal, reflecting the functional impairment of patients with cartilage injuries.

Our cohort consisted of relatively young patients (on average 32 years old at baseline) with a cartilage injury that was classified as a full-thickness focal injury at arthroscopy. Patients with a knee defect classified as general osteoarthritis at arthroscopy were excluded from our study. Even so, the radiographic examinations showed substantial osteoarthritic changes in affected knees 6 years after baseline surgery, even in groups with isolated cartilage injuries (without additional ligament or meniscal injuries), and suggest that there may be a relationship between focal cartilage injuries and early development of osteoarthritis. This is in accordance with the findings from a study by Knutsen et al. (2007), who reported osteoarthritis in one third of their patients 5 years after ACI or microfracturing. An association has also been shown between OCD diagnosed in patients after closure of the epiphyseal plates (average age 29 years old) and osteoarthritis later in life (average age 62 years old) (Linden 1977). In a regression analysis, we found that a high BMI was associated with a high Kellgren-Lawrence grade, which is consistent with results from several other studies (Spector et al. 1994, Hochberg et al. 1995, Felson et al. 1997). We did not obtain long radiographs from hip to ankle; thus, we could not measure alignment. Consequently, we could not assess a possible additional effect of malalignment on the side-to-side differences shown.

Our patients with cartilage defects were heterogeneous with respect to knee scoring and co-morbidites. However, this has also been true in published RCTs, which have shown a wide range in knee scores—both preoperatively and postoperatively—and a substantial percentage of patients with additional co-morbidities (Horas et al. 2003, Knutsen et al. 2004, 2007, Saris et al. 2008, 2009). Thus, our follow-up study reflects the clinical variation observed in patients with cartilage defects.

One limitation of our study is that, despite a high follow-up rate (84 of 96 patients; 87%), the number of patients was low. Moreover, half of them had additional injuries that caused difficulties in the interpretation of the results. In addition, several patients had had surgery in the same knee, before (42 patients) and/or after (32 patients) baseline arthroscopy. Another limitation is the use of the non-validated ICRS questionnaire. This had been published (International Cartilage Repair Society 1998) about the time that the baseline study was planned. At that time, no validated outcome scores existed for patients with cartilage injuries, and the ICRS questionnaire was regarded as a new and improved tool for the evaluation of patients with cartilage injuries. Thus, to supplement the ICRS data, we included other, validated questionnaires and a general health score (SF-36) at the follow-up. The categorization of patients into subgroups was intended to give a better description of the cohort, but the subgroups were too small to be compared with each other statistically. In any case, a comparison would have been of limited value due to several confounding factors. Thus, a regression analysis to detect possible predictors and to correct for confounding factors was performed.

Our results should remind surgeons involved in cartilage repair to question whether non-operative treatment modalities, such as active rehabilitation, might be sufficient. At present, in our institution, all patients who are candidates for cartilage surgery studies are enrolled in a 3-month physical training program study before surgery. To date, many patients have improved on this program and have decided to postpone surgery. Several RCTs have been conducted to evaluate the efficacy of surgical cartilage repair methods (Bentley et al. 2003, Horas et al. 2003, Knutsen et al. 2004, Gudas et al. 2005, Knutsen et al. 2007, Saris et al. 2008, 2009). In addition, many case series have been conducted. To our knowledge, however, no studies have included a control group of patients who received non-surgical or no treatment. In an RCT that compared mosaic plasty and ACI, where debridement of the lesion was performed at the time of enrollment, one third of the patients improved after the initial debridement and further cartilage surgery was not needed (Dozin et al. 2005). In a report on 28 patients with a cartilage defect in the knee diagnosed at arthroscopy, 22 patients functioned well 14 years after diagnosis (Messner and Maletius 1996). In that study, Pridie drilling in 3 patients and cartilage shaving or removal of free bodies in some patients were performed initially. During follow-up 5 of the patients underwent arthroscopy, and of these 3 had removal of free bodies. These findings, together with the results from the present study, suggest that non-operative treatment or less invasive surgery may be sufficient to relieve symptoms for many patients with knee cartilage defects.

In summary, we found that improvements in functional scores were possible without cartilage surgery in many patients with knee cartilage defects. However, knee function remained seriously affected. This study was not designed to compare operative and non-operative treatments of cartilage lesions. Further studies are needed to determine whether cartilage surgery can yield better functional outcomes than non-surgical or less invasive surgical treatments. We suggest that a control group of patients receiving non-surgical treatment should be included in future RCTs.

## Supplementary Appendix

Click here for additional data file.
