# Supplementary material for: 6-year follow-up of 84 patients with cartilage defects in the knee: Knee scores improved but recovery was incomplete
Source: Acta Orthop. 2010 Oct 8;81(5):611–8. doi: 10.3109/17453674.2010.519166 (PMC3214752; doi:10.3109/17453674.2010.519166)
Supplement: Supplementary file 1 [file ORT-1745-3674-81-611-s3813.pdf]

Supplementary article data

6-year follow-up of 84 patients with cartilage defects in the knee

Knee scores improved but recovery was incomplete

Sverre Løken[1,3], Stig Heir[2,3], Ingar Holme[3], Lars Engebretsen[1,3], and Asbjørn Årøen[1,3]

[1]Orthopaedic Department, Oslo University Hospital; [2]Martina Hansens Hospital; [3]Oslo Sports Trauma Research Center, Oslo, Norway
Correspondence SL: s-loek@online.no
Submitted 09-11-11. Accepted 10-03-26

Table 4. Various tests conducted at follow up to assess knee function. Patients were grouped based on the types of procedures performed

	Subgroup 1	Subgroup 2	Subgroup 3	Subgroup 4	All patients
Cartilage repair: [a]	No	No	Yes	Yes	
Ligament/meniscus surgery:	No	Yes	No	Yes	
	(n = 19/25)	(n = 35/39)	(n = 18/21)	(n = 12/13)	(n = 84/98) [b]
	Mean (SD)	Mean (SD)	Mean (SD)	Mean (SD)	Mean (SD)
Lysholm	71 (23)	83 (16)	75 (24)	67 (24)	76 (21)
Tegner [c]	3.7 (2.2)	4.9 (1.6)	4.4 (1.6)	3.8 (2.5)	4.3 (1.9)
KOOS Pain	72 (22)	82 (19)	80 (21)	72 (29)	78 (22)
KOOS Symptoms	72 (22)	78 (20)	76 (17)	67 (24)	75 (21)
KOOS Activities of daily life	83 (20)	89 (17)	87 (16)	79 (26)	86 (19)
KOOS Sport and recreation	55 (34)	68 (28)	63 (33)	48 (39)	61 (33)
KOOS Knee-related quality of life	55 (27)	64 (27)	64 (24)	54 (28)	61 (26)
IKDC	60 (24)	70 (21)	69 (24)	59 (29)	66 (23)
Cincinnati	67 (27)	76 (17)	71 (25)	62 (28)	71 (23)
SF36 Physical Function	71(22)	83 (17)	80 (23)	70 (28)	78 (22)
SF36 Role Physical	75 (25)	86 (22)	92 (14)	73 (32)	83 (24)
SF36 Bodily Pain	62 (24)	78 (24)	73 (26)	65 (33)	71 (26)
SF36 General Health	72 (20)	81 (19)	75 (21)	77 (24)	77 (20)
SF36 Vitality	58 (20)	71 (18)	66 (22)	61 (24)	65 (21)
SF36 Social Function	88 (14)	91 (15)	93 (15)	84 (23)	90 (16)
SF36 Role Emotional	94 (12)	93 (14)	97 (6)	91 (17)	94 (13)
SF36 Mental Health	86 (10)	84 (12)	87 (7)	83 (15)	85 (11)
Single leg jump: [d]					
1. > 90 %	9	15	6	5	35
2. 76–90%	1	7	3	2	13
3. 50–75%	1	4	1	1	7
4. < 50%	0	3	0	1	4

[a] Cartilage repair was defined as: an autologous chondrocyte implantation (with a preceding biopsy), an osteochondral cylinder transfer, microfracture, or fixation of osteochondral fragment.
[b] Refers to the number of patients who attended follow up in relation to the number of patients at baseline.
[c] Tegner activity score: Categorical variable 1-10 (10= highest level).
[d] Single leg jump: Categorical variable 1–4 (average one-leg jump distance with affected knee/distance with contra-lateral knee = 100%). All other scores are numeric variables 0–100 (100 = best score).
Abbreviations: KOOS= Knee injury and Osteoarthritis Outcome Score; ADL= activities of daily life; QOL=quality of life; IKDC= International Knee Documentation Committee's Subjective Knee Form; Cincinnati= Cincinnati Knee Rating System; SF36= Short Form 36 Health Survey.

Open Access - This article is distributed under the terms of the Creative Commons Attribution Noncommercial License which permits any noncommercial use, distribution, and reproduction in any medium, provided the source is credited.
DOI 10.3109/17453674.2010.519166

Table 5. Linear regression analysis shown for the main outcome variable: "Change in ICRS functional score". First a univariate regression was made for the following possible predictors at baseline: area of the cartilage lesion, location of the cartilage lesion, additional ligament or meniscus surgery and cartilage surgery. Then a multivariate analysis was made for these 4 variables combined. Finally, a second multivariate analysis was made adjusting for age at the start of symptoms, age at baseline, sex and BMI

	Univariate					Multivariate (first 4 variables)					Multivariate (all variables)				
	B	95% CI		p-value	R^2	B	95% CI		p-value	R2	B	95% CI		p-value	R^2
Possible predictors:															
Area of cartilage lesion	-0.038	-0.27	0.20	0.7	0.001	-0.075	-0.33	-0.18	0.6	0.015	-0.052	-0.34	0.24	0.7	0.017
Location of cartilage lesion	-0.026	-0.16	0.11	0.7	0.002	-0.011	-0.15	0.13	0.9		0.016	-0.14	0.17	0.8	
Ligament or meniscus surgery	-0.014	-0.50	0.47	1.0	<0.001	0.012	-0.50	0.52	1.0		-0.012	-3.24	-3.24	1.0	
Cartilage repair	0.205	-0.29	0.71	0.4	0.009	0.256	-0.30	0.82	0.4		0.204	-0.34	0.24	0.5	
Possible confounding factors:															
Age at the start of symptoms											-0.007	-0.14	0.17	0.7	
Age at baseline											0.008	-0.61	0.58	0.7	
Sex											-0.169	-0.40	0.81	0.6	
Body Mass Index											0.018	-0.05	0.04	0.6	
